# Crystal structure of *Saccharomyces cerevisiae *6-phosphogluconate dehydrogenase Gnd1

**DOI:** 10.1186/1472-6807-7-38

**Published:** 2007-06-14

**Authors:** Weiwei He, Yi Wang, Wei Liu, Cong-Zhao Zhou

**Affiliations:** 1 Hefei National Laboratory for Physical Sciences at Microscale, and School of Life Sciences, University of Science and Technology of China, Hefei, Anhui, 230027, People's Republic of China

## Abstract

**Background:**

As the third enzyme of the pentose phosphate pathway, 6-phosphogluconate dehydrogenase (6PGDH) is the main generator of cellular NADPH. Both thioredoxin reductase and glutathione reductase require NADPH as the electron donor to reduce oxidized thioredoxin or glutathione (GSSG). Since thioredoxin and GSH are important antioxidants, it is not surprising that 6PGDH plays a critical role in protecting cells from oxidative stress. Furthermore the activity of 6PGDH is associated with several human disorders including cancer and Alzheimer's disease. The 3D structural investigation would be very valuable in designing small molecules that target this enzyme for potential therapeutic applications.

**Results:**

The crystal structure of 6-phosphogluconate dehydrogenase (6PGDH/Gnd1) from *Saccharomyces cerevisiae *has been determined at 2.37 Å resolution by molecular replacement. The overall structure of Gnd1 is a homodimer with three domains for each monomer, a Rossmann fold NADP^+ ^binding domain, an all-α helical domain contributing the majority to hydrophobic interaction between the two subunits and a small C-terminal domain penetrating the other subunit. In addition, two citrate molecules occupied the 6PG binding pocket of each monomer. The intact Gnd1 had a *Km *of 50 ± 9 μM for 6-phosphogluconate and of 35 ± 6 μM for NADP^+ ^at pH 7.5. But the truncated mutants without the C-terminal 35, 39 or 53 residues of Gnd1 completely lost their 6PGDH activity, despite remaining the homodimer in solution.

**Conclusion:**

The overall tertiary structure of Gnd1 is similar to those of 6PGDH from other species. The substrate and coenzyme binding sites are well conserved, either from the primary sequence alignment, or from the 3D structural superposition. Enzymatic activity assays suggest a sequential mechanism of catalysis, which is in agreement with previous studies. The C-terminal domain of Gnd1 functions as a hook to further tighten the dimer, but it is not necessary for the dimerization. This domain also works as a lid on the substrate binding pocket to control the binding of substrate and the release of product, so it is indispensable for the 6PGDH activity. Moreover, the co-crystallized citrate molecules, which mimic the binding mode of the substrate 6-phosphogluconate, provided us a novel strategy to design the 6PDGH inhibitors.

## Background

The 6-phosphogluconate dehydrogenase (6PGDH, *EC 1.1.1.44*) is the third enzyme of the oxidative branch of the pentose phosphate pathway. This pathway has two major functions: the production of ribulose 5-phosphate which is required for the nucleotide synthesis, and the generation of NADPH which provides the major reducing power essential for protecting the cell against oxidative stress and a variety of reductive biosynthetic reactions, particularly lipid production. Both thioredoxin reductase and glutathione reductase require NADPH as the electron donor to reduce oxidized thioredoxin or glutathione (GSSG)[1]. Since thioredoxin and GSH are important antioxidants[2], it is not surprising that 6PGDH plays a critical role in protecting cells from oxidative stress. Recently there is renewed interest in this pathway since it has been shown to play a central role in tumor proliferation process[3,4]. The 6PGDH catalyzes the oxidative decarboxylation of 6-phosphogluconate (6PG) to ribulose 5-phosphate (Ru5P) and CO_2 _with the concomitant reduction of NADP^+ ^to NADPH[5]. This reaction is similar to those catalyzed by isocitrate dehydrogenase (IDH) and malic enzyme because all three yield a ketone, CO_2_, and NAD(P)H as products. However, unlike the other enzymes, 6PGDH is metal-ion independent[6].

The enzyme 6PGDH has been reported to be involved in several human diseases, including cancer[7] and Alzheimer's disease(AD)[8], through various studies over the last three decades [9-11]. One of the correlations between 6PGDH and these diseases is oxidative stress. As we all know cancer is a genetic disease. Oxidative stress induces DNA damage including modified base products and strand breaks that may lead to further mutation and chromosomal aberration of cancer. Although it remains an open question as to whether oxidative stress is a causative factor or a consequence of AD, the correlation between oxidative stress and AD is well established[12]. Many evidence suggested that increased oxidative damage is an early event in AD[13]. As a compensatory response to elevated brain oxidative stress, the activities of 6PGDH were increased in AD[8].

**Figure 1:**
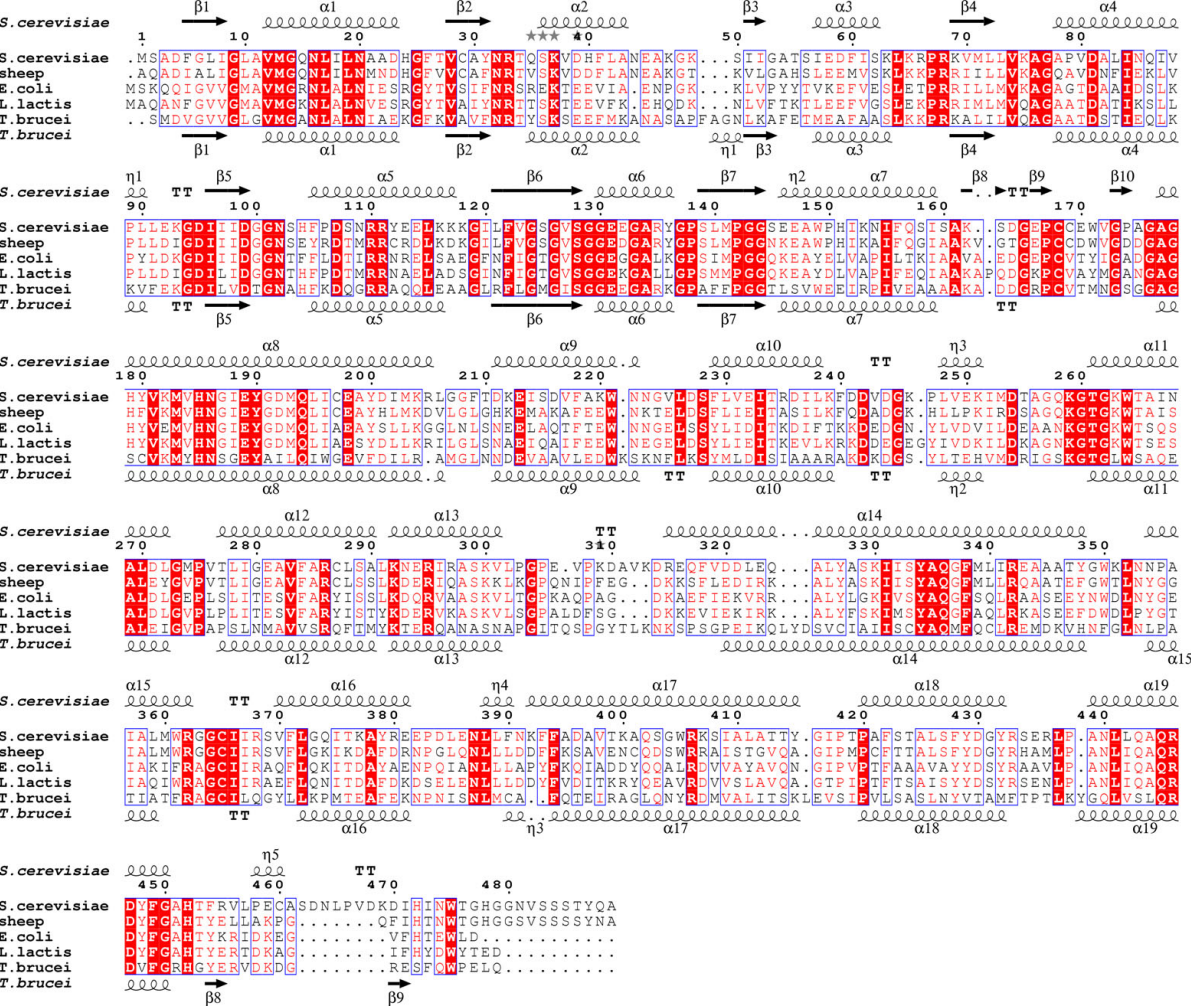
**Multiple alignment of 6-phosphogluconate dehydrogenases from *Saccharomyces cerevisiae*, sheep, *Escherichia coli, Lactococcus lactis *and *Trypanosoma brucei***. All sequences were obtained from NCBI databases and alignments were performed using the programs MultAlin [15] and ESPript [16].

Prokaryotic and eukaryotic 6PGDHs are generally homodimers, with a monomer of ~470 amino acids and a molecular weight of ~52 kDa[14]. Each subunit is comprised of an N-terminal Rossmann fold coenzyme-binding domain, a large all-helical domain and a small C-terminal tail. The active dimer assembles with the C-terminal tail of two subunits threading through each other. The coenzyme binding domain of 6PGDH has an α-β-α fold, while the substrate 6PG was located in the cleft between the α helices of one subunit and the C-terminal tail of the other subunit of the dimer. As expected from their essential biological functions, the amino acid sequences of 6PGDHs from various organisms show significant conservation. The complete 6PGDH sequences from five different species, including *Saccharomyces cerevisiae*, sheep, *Escherichia coli*, *Lactococcus lactis *and *Trypanosoma brucei*, were aligned using the programs MultAlin[15] and ESPript[16] to show the conservation among species (Figure 1).

The kinetic mechanism of the enzymes from sheep liver[6,17-20] and from *Candida utilis*[21] has been studied in detail. A considerable number of factors are capable of modifying the catalytic activity of 6PGDH. From the sheep liver 6PGDH, the oxidative decarboxylation reaction was reported as asymmetric in terms of ordered product release: carbon dioxide first and NADPH last, while the favored binding order for NADP^+ ^and 6PG is dependent on the buffer system used. In phosphate buffer, it seems that NADP^+ ^binds to the enzyme before 6PG[6], but in triethanolamine (TEA) buffer at pH 7.0 it seems that an initial complex composed of the enzyme and 6PG is dominant. In either buffer product release is in the same order with carbon dioxide leaving first, followed by Ru5P. Multiple isotope effects have been used to interpret the sequential mechanism[22]. However, a recent investigation under a wider range of conditions suggests an acid-base mechanism[23,24]. The general base accepts a proton from the 3-hydroxyl group of 6PG concomitant with hydride transfer and then shuttles the proton between itself and the sugar oxygen throughout the reaction, ultimately accepting it as ribulose is formed. The general acid presumably plays a role in only the last of the three steps, namely, the tautomerization of the enediol of ribulose 5-phosphate to the keto product[25].

Due to the potential importance of 6PGDH in human diseases and medicine (i.e., development of selective inhibitors for therapeutic approaches), it is crucial to better understand its molecular function through the 3D structural studies of this enzyme from multiple species[26]. So far 6PGDH crystal structures have been solved in the three species (sheep[27,28], *T. brucei*[29] and *L. lactis*[30]). Since yeasts are comparatively similar in structure to human cells, both being eukaryotic, in contrast to the bacteria and archaea, we examined the structural and biochemical characteristics of 6PGDH in *S. cerevisiae*, which is one of the most intensively studied eukaryotic model organisms.

In *S. cerevisiae*, the open reading frame *YHR183W/GND1 *encodes the major isoform of the two 6PGDHs, named Gnd1, accounting for approximately 80% of the total 6PGDH activity, whereas Gnd2 encodes the minor isoform[31]. Gnd1 displays very high similarity to Gnd2 (86% identity). In this study we overexpressed, purified and characterized Gnd1 in *E. coli*. Moreover, we determined the crystal structure of Gnd1 in complex with two citrate molecules by molecular replacement and refined it to 2.37 Å resolution. Based on the comparative structural analyses in combination with the enzymatic kinetics studies, we obtained more insights into the molecular mechanism of this enzyme, especially the structure-based function of the C-terminal domain.

## Results and discussion

### Overall structure

**Figure 2:**
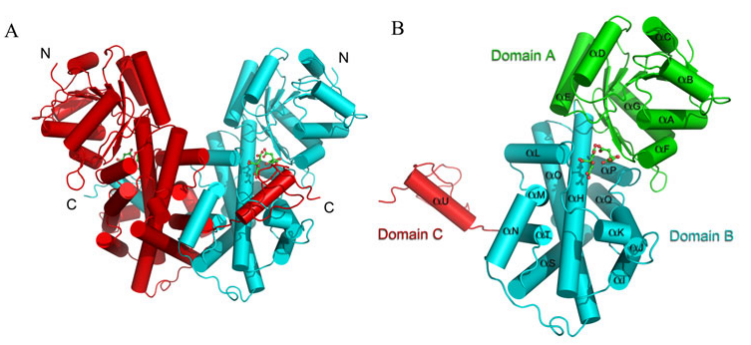
**The overall structure and organization of Gnd1**. **(A) **The cartoon representation of Gnd1 homodimer. The citrate molecules are shown in sticks and colored according to atom types, C is green and O is red. **(B) **The Gnd1 monomer contains three domains, domain A, B and C colored in green, cyan and red, respectively. The figures were made using PyMOL [34].

The overall structure of the dimer of *S. cerevisiae *6PGDH/Gnd1 enzyme with two molecules of citrate is illustrated in Figure 2A. The structure of Gnd1 (PDB code: 2P4Q) was determined by molecular replacement using sheep 6PGDH as the starting model (PDB code: 1PGP). The structure was determined at 2.37 Å resolution. The final model of each monomer contains residues 1–476, two citrate molecules and 212 water molecules (Table 1).

**Table 1:** Data Collection and Refinement Statistics.

Data collection	
Space group	*P*6_5_22
Unit-cell parameters (Å)	a = b = 147.26, c = 114.42,α=β = 90°, γ = 120°
Wavelength (Å)	1.5418
Total reflections	347,811
Unique reflections	29,518
Completeness (%), overall/outer shell	96.9% (94.0%)
Resolution range (Å), overall/outer shell	21.32–2.37 (2.45–2.37)
Refinement statistics	
Rmerge^a ^(%) overall/outer shell	7.41 (36.29)
R-factor^b ^(%)	20.8
R-free^c ^(%)	21.8
Number of atoms	
Protein	3,683
Water	212
Citrate molecule	32
Rms deviation from target^d^	
Bond lengths (Å)	0.009
Bond angles (°)	1.3
Average B factors:	
Protein atoms	49.1
Water atoms	52.8
Citrate atoms	61.7
Ramachandran plot (%)^e^	91.7/7.6/0.7/0

^a ^Rmerge=∑h∑i|Ihi−〈Ih〉|∑h∑iIhi
 MathType@MTEF@5@5@+=feaafiart1ev1aaatCvAUfKttLearuWrP9MDH5MBPbIqV92AaeXatLxBI9gBaebbnrfifHhDYfgasaacH8akY=wiFfYdH8Gipec8Eeeu0xXdbba9frFj0=OqFfea0dXdd9vqai=hGuQ8kuc9pgc9s8qqaq=dirpe0xb9q8qiLsFr0=vr0=vr0dc8meaabaqaciaacaGaaeqabaqabeGadaaakeaacqWGsbGudaWgaaWcbaGaemyBa0MaemyzauMaemOCaiNaem4zaCMaemyzaugabeaakiabg2da9maalaaabaWaaabeaeaadaaeqaqaamaaemaabaGaemysaK0aaSbaaSqaaiabdIgaOjabdMgaPbqabaGccqGHsisldaaadeqaaiabdMeajnaaBaaaleaacqWGObaAaeqaaaGccaGLPmIaayPkJaaacaGLhWUaayjcSdaaleaacqWGPbqAaeqaniabggHiLdaaleaacqWGObaAaeqaniabggHiLdaakeaadaaeqaqaamaaqababaGaemysaK0aaSbaaSqaaiabdIgaOjabdMgaPbqabaaabaGaemyAaKgabeqdcqGHris5aaWcbaGaemiAaGgabeqdcqGHris5aaaaaaa@536F@, where I_hi _is the i^th ^observation of the reflection h, while I_h _is the mean intensity of reflection h.

^b ^Rfactor=∑||Fobs|−|Fcalc|||Fobs|
 MathType@MTEF@5@5@+=feaafiart1ev1aqatCvAUfKttLearuWrP9MDH5MBPbIqV92AaeXatLxBI9gBaebbnrfifHhDYfgasaacH8akY=wiFfYdH8Gipec8Eeeu0xXdbba9frFj0=OqFfea0dXdd9vqai=hGuQ8kuc9pgc9s8qqaq=dirpe0xb9q8qiLsFr0=vr0=vr0dc8meaabaqaciaacaGaaeqabaqabeGadaaakeaacqWGsbGudaWgaaWcbaGaemOzayMaemyyaeMaem4yamMaemiDaqNaem4Ba8MaemOCaihabeaakiabg2da9maaqaeabaWaaSaaaeaadaabdaqaamaaemaabaGaemOray0aaSbaaSqaaiabd+gaVjabdkgaIjabdohaZbqabaaakiaawEa7caGLiWoacqGHsisldaabdaqaaiabdAeagnaaBaaaleaacqWGJbWycqWGHbqycqWGSbaBcqWGJbWyaeqaaaGccaGLhWUaayjcSdaacaGLhWUaayjcSdaabaWaaqWaaeaacqWGgbGrdaWgaaWcbaGaem4Ba8MaemOyaiMaem4CamhabeaaaOGaay5bSlaawIa7aaaaaSqabeqaniabggHiLdaaaa@5854@, where *F*_*obs *_and *F*_*calc *_are the observed and calculated structure factor amplitudes, respectively.

^c ^R_free _was calculated with a small fraction (5%) of randomly selected reflections. No refinement was done on the 5% of randomly selected reflections at any stage.

^d ^Root-mean-square deviation of bond lengths or bond angles from ideal geometry.

^e ^Percentage of residues in most favoured/additionally allowed/generously allowed/disallowed regions of Ramachandran plot, according to PROCHECK.

**Figure 3:**
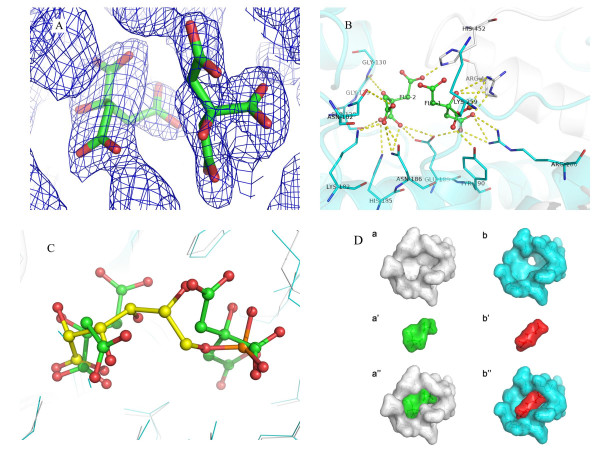
**The binding mode of the two citrate molecules**. **(A) **Electron density of the two citrate molecules FLC1 and FLC2 (*2Fo-Fc *map contoured at 1.2 σ). **(B) **A closer look of the conserved residues binding to the two citrate molecules. The C terminal tail of chain B is colored in grey, and chain A in cyan. **(C) **Superimposed structures of Gnd1 (in cyan) with sheep liver 6PGDH (PDB code: 1PGP; colored in grey). The two citrate molecules (shown in sticks) are superimposed on one molecule of 6PG (shown in sticks) of 1PGP. **(D) **The surface comparison between yeast Gnd1 bound to two citrate molecules (a, a' and a") and sheep liver 6PGDH monomer bound to 6PG (b, b' and b"). The monomer omitting the bound ligand, the ligand and the complex are shown in a/b, a'/b' and a"/b", respectively.

All the calculations of rotation function and translation function were conducted using the program MOLREP[32] in CCP4 (Correlation coefficient: 53.5%). Refinement was carried out using the programs O and crystallography and NMR system (CNS)[33]. Through the refinement we identified two unexpected electron clouds in the catalytic pocket as citrate molecules used in crystallization. It appeared that two citrate molecules were bound to the enzyme in each monomer (Figure 2A[34], for more details see Figure 3A).

Similar to the 6PGDHs reported in other species, Gnd1 forms dimer. Each subunit has three domains (A, B and C in Figure 2B). Domain A includes a typical dinucleotide binding motif-"Rossmann Fold" (residues 1 to 127) and two additional α/β units (residues 128 to 175) which form the N-terminal α/β "co-enzyme binding" domain. This domain exhibits high similarity to other NADP^+ ^binding domains such as the 3-hydroxyisobutyrate dehydrogenase (HIBADH) from *Thermus thermophilus *HB8[35], with a root-mean-square deviation (rmsd) of 2.3 Å. This data substantiates previous phylogenetic evidence that 6PGDH and HIBADH may share a common evolutionary origin and enzymatic mechanism[36]. Domain B consists of residues 176–433 and is almost exclusively helical in structure(α_H_-α_T_). Domain C (residues 434 to 476) contains one helix (α_U_) and one loop (Figure 2B). The two all-α domains contain most of the residues involved in substrate binding and dimerization. The dimer is formed by the C-terminal tail of two subunits threading through each other forming a mobile lid on the substrate binding pocket.

The penetration of the third domain through the other monomer in the biological unit indicates a concerted folding pathway of the monomers during the translational or post-translational process. Although the mean average temperature factor of Gnd1 is as high as 49.1, we still found a difference of temperature factor among the three domains, which are 58.0, 42.5 and 53.5 respectively. This is consistent with previous observations from the structures of 6PGDH from sheep liver and protozoan parasite, which showed the first domain's higher mobility in the absence of dinucleotide co-enzyme[28,29]. Gnd1 and 6PGDH of sheep liver both have smaller dimer interface of around 5500 Å^2 ^compared to the 6PGDH of *T. brucei*, which is around 6300 Å^2^. This is likely due to fewer residues (109 and 115 vs 134 amino acids) involved in the monomer-monomer interactions of the yeast Gnd1 and sheep 6PDGH[29].

Through the analysis of the surface potential, it is obvious that dimerization is not completely due to the interaction between the C-termini of each monomer. In fact, the interactions between the hydrophobic groups of Domain B of each monomer contribute the majority to the dimerization.

### The binding of two citrate molecules

To our surprise, two citrate molecules were bound to the active site of each Gnd1 monomer (Figure 3). In citrate FLC1, Oγ2 replaced the water molecule (HOH528) in the structure of 6PGDH-6PG complex (PDB code: 1PGP), hydrogen bonded to Oξ2 of Glu189 and formed a salt bridge with Nη2 of Arg286. OHβ was located within 4 Å from His452 and Arg446 of Chain B, thus its negative charge is balanced by these two residues. In citrate FLC2, Oα2 interacted electrostatically with His452 in Chain B, while Oα1 did not form any hydrogen bonds, nor salt bridges, with other residues. The role of O3 in 6PG was substituted partly by Oγ1, interacting with Asn102; and partly by Oγ2, interacting with Asn186 and Lys182. Oβ1 and Oβ2 located at the same place of O1α and O1 in 6PG respectively. And the position of O2 in 6PG was replaced by OHβ, interacting with Gly129 and Gly130.

By superimposing Gnd1 to the sheep liver 6PGDH (PDB code: 1PGP), we found that the two citrate molecules occupied the space of one molecule of 6PG (Figure 3C). Moreover, the interactions and residues involved are strikingly similar[37] (Table 2). Although citrate has as many carbon atoms as 6PG, it is highly branched which reduces its effective length to about half that of 6PG. The negative charges between two citrate molecules lead to electrostatic repulsion, so they occupy a larger space than 6PG (Figure 3D). However, this electrostatic repulsion could be compensated for by hydrogen bonds around the active site of Gnd1.

**Table 2:** Comparison of residues interacting with two citrate molecules (FLC1 and FLC2) in Gnd1 and Gluconate-6-phosphate (6PG) in sheep liver 6PGDH (PDB code: 1PGP). (Except for Arg446 and His452, all other residues are in chain A.)

**FLC1**	6PG	**FLC2**	6PG
**Glu189**	Glu190	**Asn102**	Asn102
**Tyr190**	Tyr191	**Ser128**	Ser128
**Lys259**	Lys260	**Gly129**	Gly129
**Thr261**	Thr262	**Gly130**	Gly130
**Arg286**	Arg287	**Lys182**	Lys183
**Arg446 (chain B)**	Arg446 (chain B)	**His185**	His186
**His452 (chain B)**	His452 (chain B)	**Asn186**	Asn187
		**Glu189**	Glu190

Human African trypanosomiasis (sleeping sickness) has re-emerged as a major health threat in Sub-Saharan Africa which caused by parasitic protozoan *Trypanosoma brucei*[38]. 6PGDH emerges as a potential drug target in this disease[39]. Previous study has shown that citrate serves as an inhibitor of 6PGDH[40]. Structural studies of 6PGDH will definitely facilitate the development of 6PGDH inhibitors for potential therapeutic use. Our current research provides for the first time the conformation of 6PGDH bound with an inhibitor.

### The mutant proteins Gnd1ΔC35, Gnd1ΔC39 and Gnd1ΔC53

The yeast Gnd1 consists of 489 residues, with the residues after Ser434 forming a small C-terminal domain. Sequence comparison of the Gnd1 with the corresponding enzymes derived from sheep, *E. coli*, *L. lactis *and *T. brucei *revealed the substrate-binding residues to be identical in all these species, but the sequences of the C-terminal tail are not well conserved, especially the region of residues 457–489 (Figure 1). From the structure of Gnd1 we found two highly conserved residues, Arg446 and His452, to be vitally important for citrate binding. These two residues were also shown to be critical for 6PG binding from structural studies of sheep liver 6PGDH[28]. The residue Arg446 was reported to play an important role in anchoring substrate while 6PG is oxidatively decarboxylated to ribulose 5-phosphate[41]. In an attempt to understand the potential function of the C-terminal tail on the dimerization and enzymatic activity we generated mutant Gnd1 with C-terminal 35, 39 and 53 amino acids deletions. These proteins, designated Gnd1ΔC35, Gnd1ΔC39 and Gnd1ΔC53, which contain residues 1–454, 1–450 and 1–436, were overexpressed and purified, respectively. Gnd1ΔC35 contains both conserved residues (Arg446 and His452), while Gnd1ΔC39 contains only Arg446, and Gnd1ΔC53 is a complete deletion of the entire C-terminal tail (Figure 3B).

Despite the fact that all of the truncated enzymes were soluble and could be purified by Ni^2+ ^chromatography and gel filtration using a Superdex™ 200 column, none of them had detectable enzymatic activity (data not shown). It suggests that the intact C-terminal tail is vitally important for the enzymatic activity, even the region of residues 454–489 is not conserved among different species, nor directly interacting to the substrate 6PG or its homologs.

In addition, through purification and crystallization we found that Gnd1ΔC53 is less stable than the full length Gnd1, although the mutant proteins still dimerized. Obviously the dimerization was not completely sustained by the C-terminal tail. This is the first data to suggest that the C-terminal tail of 6PGDH is dispensable for dimerization. In fact, the interactions of the hydrophobic groups of the Domain B, which are mostly composed of helices, are the major driving force for the dimer formation. However, the C-terminal tail contributes a part to maintaining the stability of the protein.

### Km values for the Gnd1

**Figure 4:**
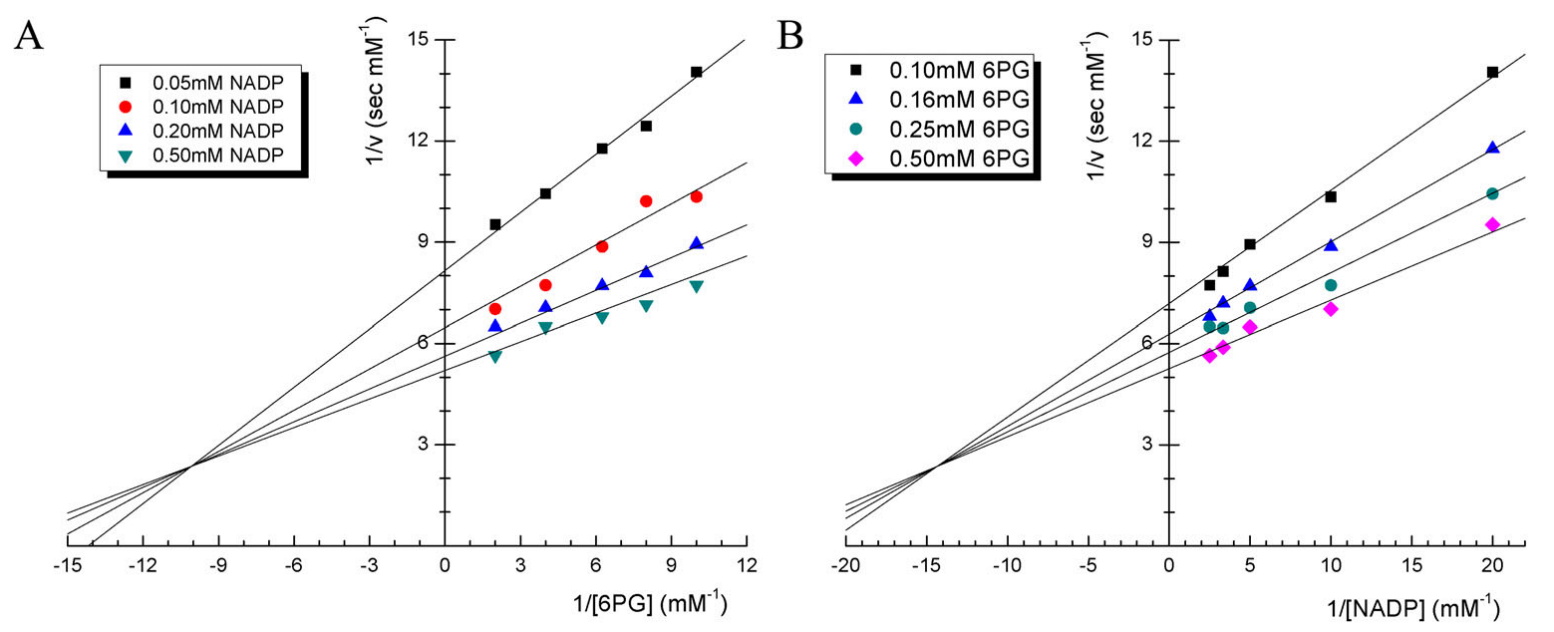
**Lineweaver-Burk plots of initial-rate measurements of Gnd1**. The kinetic parameters of Gnd1 were obtained by varying the concentration of each substrate (in the range 0.1–0.5 mM for 6PG; 0.05–0.4 mM for NADP^+^). **(A) **Measurements as a function of 6PG concentration. **(B) **Measurements as a function of NADP^+ ^concentration.

Detailed kinetic analyses of the 6PGDHs from *T. brucei*[42], *L. lactis*[41] and sheep[18] have previously been performed. Earlier studies showed 6PGDHs from different species exhibit very similar binding mechanism with the natural substrate, with only minor differences in the *Km *for the substrate and the coenzyme[43]. As we know, sequential reactions (both random and ordered) are characterized by lines that intersect to the left of the 1/v axis in Lineweaver-Burk double-reciprocal plot, while in Ping-Pong reactions the lines parallel. To further validate the previous findings, we determined the *Km *values of Gnd1 at pH 7.5 and the ionic strength of 0.03 (Figure 4). The kinetic parameters were determined by varying the concentration of each substrate (in the range 0.1–0.5 mM for 6PG; 0.05–0.4 mM for NADP^+^). It is obvious that in each plot there was an intercept to the left of the 1/v axis, as calculated from Figure 4A and 4B. Double-reciprocal plots of enzyme rate measurements as a function of substrate concentration indicate *Km *values of 50 ± 9 μM for 6PG, and 35 ± 6 μM for NADP^+ ^at pH 7.5. The initial velocity pattern of Gnd1 intersects to the left of the ordinate, suggesting a sequential kinetic mechanism which is in agreement with that of the enzymes from other species. Kinetic analysis of this enzyme would also indicate the same mechanism of oxidative decarboxylation as in the sheep liver enzyme[23].

## Conclusion

The 6PGDH is an important enzyme of the pentose phosphate pathway and has been linked to several human diseases. Here we provide the X-ray structure of *S. cerevisiae *6PGDH/Gnd1. The tertiary structure of Gnd1 exhibits a high similarity to 6PGDH in other species, as well as conserved substrate and coenzyme binding residues. Kinetic studies suggest a sequential mechanism for Gnd1. However, our structure reveals for the first time the binding mode of two inhibitory citrate molecules in the Gnd1 substrate binding pocket, which provides clues for the development of specific inhibitors against 6PGDH. We further studied the role of the Gnd1 C-terminal tail and found that it is dispensable for dimerization, but crucial for the enzymatic activity.

## Methods

### Cloning, expression and purification

The *YHR183W/GND1 *gene was amplified by PCR using the genomic DNA of *S. cerevisiae *strain S288C as the template. An additional sequence coding for a six-histidine tag was introduced at the 5' end of the gene during PCR amplification. The PCR product was then cloned into a pET28a-derived vector between *Nde *I and *Not *I restriction sites. Expression was done at 37°C using the transformed *E. coli *Rossetta (DE3) strain and 2 × YT medium (OXOID LTD.) supplemented with kanamycin at 30 μg/ml and chloramphenicol at 34 μg/ml. When the cell culture reached an OD_600nm _of 0.6, protein expression was induced with 0.2 mM IPTG (BBI) and the cells were grown for a further 4 hrs. Cells were collected by centrifugation, suspended in 30 ml buffer containing 20 mM Tris-HCl, pH 8.0, 200 mM NaCl, 20 mM β-mercaptoethanol and stored overnight at -20°C. Cells were lysed by three cycles of freezing/thawing and sonication. The His-tagged proteins were purified using a Ni^2+ ^affinity column with standard protocols. Eluted protein was further purified by gel filtration using a Superdex™ 200 column (Amersham Biosciences) equilibrated in 20 mM Tris-HCl, pH 8.0, 200 mM NaCl and 20 mM β-mercaptoethanol. The purity of the pooled fractions was checked by SDS-PAGE and the integrity of the protein samples was confirmed by mass spectrometry.

The DNA sequences of *GND1 *without the sequence coding for the C-terminal 35, 39 and 53 residues (Gnd1ΔC35, Gnd1ΔC39 and Gnd1ΔC53) were amplified, respectively. PCR products were purified using the DNA gel extraction kit (V-gene, China) and inserted into pET28a-derived vector. The mutant proteins (Gnd1ΔC35, Gnd1ΔC39 and Gnd1ΔC53) were overexpressed and purified respectively as described above.

### Crystallization of Gnd1

Crystals of Gnd1 were obtained at 15°C by the hanging-drop vapour diffusion method. For crystallization, the protein concentration was 17.3 mg/ml, in a buffer containing 20 mM Tris-HCl, pH 8.0, 50 mM NaCl and 20 mM β-mercaptoethanol. In each drop, 1μl of the protein solution was mixed with 1μl of the reservoir solution and the mixture was equilibrated against 0.5 ml of the reservoir solution (1.28 M tri-Sodium Citrate at pH 6.5). Crystals with a maximal size of 100–200 μm appeared within 3 days. For data collection, the crystals were frozen in liquid nitrogen after soaking in cryoprotectant buffer containing 30% glycerol and 1.28 M tri-Sodium Citrate at pH 6.5.

### Data collection and structure determination

The crystal was flash frozen in a stream of nitrogen gas to 110 K. In total 102 images of diffraction data were collected using MAR345dtb detector (MarResearch, Germany), with wavelength of 1.5418 Å and oscillation of 1 degree. X-ray crystallographic data were processed using AUTOMAR. The structure was determined by molecular replacement with the program MOLREP[32] using the structure of 6-PGDH (PDB code: 1PGP) as the initial model. Crystallographic refinement was performed using programs O and CNS[33]. The final model consists of residues 1–476 for one monomer that are visible within the electron density and 212 water molecules. Structure factors and the coordinates have been deposited in the PDB (code: 2P4Q). The final statistics and refinement parameters are listed in Table 1.

### Enzymatic activity assays

The assays were performed at 28°C by measuring the initial rate in the direction of oxidative decarboxylation of 6PG. To calculate the specific activity, NADPH production was measured at 340 nm in a MODEL U-2800 UV-VIS spectrophotometer (HITACHI). The enzymes containing 30% glycerol were conserved at -80°C. The standard enzyme assay solution of 200 μl contained (final concentrations): Gly-Gly buffer (50 mM, final pH7.5); MgCl_2 _(10 mM); NADP^+ ^(0.6 mM); 6PG (2 mM) and enzyme (4.8 nM). All buffers used were prepared with deionized and distilled water. All assays were performed at least in duplicate; means of replicates were used as single points in subsequent statistical analyses. The enzymatic activity of Gnd1, Gnd1ΔC35, Gnd1ΔC39 and Gnd1ΔC53 was measured respectively, as described above.

### Determination of kinetic parameters

The catalytic activities of Gnd1 were assayed by measuring the absorbance of NADPH at 340 nm, as described above. The reaction rate (v) and substrate concentration were plotted in a double reciprocal manner to calculate the kinetic parameters. The kinetic parameters of wild-type enzyme Gnd1 were determined by varying the concentration of each substrate (in the range 0.1–0.5 mM for 6PG; 0.05–0.4 mM for NADP^+^) for five fixed concentrations of the other. The assays initiated by the addition of the enzyme. Values were then calculated from Lineweaver-Burk plots with the respective slope and intercept replots. The slopes of the lines were drawn as the best fit to the experimental points.

1v=1Vmax⁡(KmA+KsAKmB[B])1[A]+1Vmax⁡(1+KmB[B])
 MathType@MTEF@5@5@+=feaafiart1ev1aqatCvAUfKttLearuWrP9MDH5MBPbIqV92AaeXatLxBI9gBaebbnrfifHhDYfgasaacH8akY=wiFfYdH8Gipec8Eeeu0xXdbba9frFj0=OqFfea0dXdd9vqai=hGuQ8kuc9pgc9s8qqaq=dirpe0xb9q8qiLsFr0=vr0=vr0dc8meaabaqaciaacaGaaeqabaqabeGadaaakeaadaWcaaqaaiabigdaXaqaaiabdAha2baacqGH9aqpdaWcaaqaaiabigdaXaqaaiabdAfawnaaBaaaleaacyGGTbqBcqGGHbqycqGG4baEaeqaaaaakmaabmaabaGaem4saS0aa0baaSqaaiabd2gaTbqaaiabdgeabbaakiabgUcaRmaalaaabaGaem4saS0aa0baaSqaaiabdohaZbqaaiabdgeabbaakiabdUealnaaDaaaleaacqWGTbqBaeaacqWGcbGqaaaakeaadaWadaqaaiabdkeacbGaay5waiaaw2faaaaaaiaawIcacaGLPaaadaWcaaqaaiabigdaXaqaamaadmaabaGaemyqaeeacaGLBbGaayzxaaaaaiabgUcaRmaalaaabaGaeGymaedabaGaemOvay1aaSbaaSqaaiGbc2gaTjabcggaHjabcIha4bqabaaaaOWaaeWaaeaacqaIXaqmcqGHRaWkdaWcaaqaaiabdUealnaaDaaaleaacqWGTbqBaeaacqWGcbGqaaaakeaadaWadaqaaiabdkeacbGaay5waiaaw2faaaaaaiaawIcacaGLPaaaaaa@5D2D@

## Authors' contributions

WWH cloned, expressed, purified and crystallized the protein, and performed the activity assays. YW performed data collection, structure determination and structure-function analysis. WL refined the structure. CZZ coordinated all the components of the project, and provided financial support. WWH and CZZ wrote the paper. All authors have read and approved the final manuscript.
